# Cycling and Health

**Published:** 1891-09-26

**Authors:** 


### The Hospital

A Weekly Journal of

Science, Medicine, Nursing, and Philanthropy.

*For Hospitals, Asylums, Infirmaries, Dispensaries, Medical Practitioners, Students, Nurses, and the Charitable Public.*

Vol. X.—No. 261.] [Sept. 26, 1891.

### Cycling and Health.

Nobody can deny it: the middle-aged man, including, of course, the woman, is the true hero in life's battle. He does the toughest of the fighting; and he deserves the sweetest of the plums of victory. All the world in France has been looking on at the great cycle race between Paris and Brest. The most successful competitor was, of course, M. Charles Terront, who covered the distance, nearly seven hundred and fifty miles, in less than three days: but by far the most interesting, at least from a middle-aged man's point of view, is M. Rousset, who took six and a-half days for the feat. M. Rousset is fifty-six years of age, and is a citizen of Bordeaux. He was presented with a personal gift on the occasion by M. Marinone, of the *Petit Journal*, in honour of his striking performance.

By all middle-aged men this journey of nearly seven hundred and fifty miles in six and a-half days, that is about a hundred and fifteen miles a-day for six days in succession, by a man of fifty-six, is a thing to be well weighed and considered. M. Rousset carried himself, so to speak, all that distance by his own powers and a little cheap mechanism. Moreover, he was probably in better health at the end of his journey than he was at the beginning.

This performance of M. Rousset should be taken in conjunction with Dr. Richardson's letters on Cycling, which appeared last week in the *Daily Graphic.* Dr. Richardson is a staunch believer in the religion of Cycles. He has professed and practised his faith from the earliest days of its foundation, or rather of its modern development, until now: and he speaks with all the ardour of an enthusiast, and all the sober wisdom of an experienced professor.

"Health and happiness for the middle-aged," is the thesis we set out to prove. The chief trouble of the average middle-aged man of the city class is what is known as a "sluggish" or "torpid" liver. We need not enter into any of the niceties of pathology here: the man in the street, as well as the doctor, has a fairly clear idea in his mind of what is meant by a sluggish liver. Now the bicycle or the tricycle, when properly used, converts the sluggish into an active liver; and that in double-quick time. It is quite easy to understand why this should be so. The sluggish liver is the liver in which the blood circulates too slowly, and remains too long before it is passed forward into the lung circulation. The act of pedalling the cycle instantly quickens the circulation in the feet and legs, and in the course of a quarter or half an hour sends a perfect torrent of blood through the veins of the legs and the pelvis into the portal vein, which spreads itself out in the liver from the lower side. In other words, the effect of cycling upon the liver is the same as that of flooding a still and stagnant lake with innumerable mountain streams after a heavy rainfall. The whole of the water in the lake is violently moved, and the outflow at its lower extremity is increased tenfold. It is precisely this flooding effect which is produced upon the liver by the rushing currents of blood from the legs as the result of their energetic activity in pedalling the bicycle or tricycle. The stagnant lake in the organ is moved in every part, all its minutest ramifying vessels are washed out, its bile-secreting cells are stimulated by fresh blood; and the work which a liver pill usually takes ten or twelve hours to perform, is performed in less than one hour, and with much more thoroughness and naturalness. Not that the immediate effect of cycling is to rouse the bowels to activity: but the constipation and liver torpidity are gradually overcome by an increase of normal physiological activity: and the cure remains permanent so long as the exercise is persevered with. This at least, is the writer's personal experience; and, the physiological series of events being such as is here described, a similar experience must have resulted with many other cyclists.

The oxygenation of the blood is another of the most fundamental of all the conditions of health. This is one of the commonest of physiological commonplaces, and the man who has no practical faith in it must be, physiologically speaking, a fool. One of the earliest effects of moderate cycling is seen in the changed colour of the lips, and in the reddening tint which is soon imparted to the sallow and leaden complexion of middle age. The average town man of fifty has blue lips, very blue; and blue lips are bad lips, very bad. They denote blue blood, and blue blood is bad blood; it is blood without oxygen in it; it cannot carry off the waste which it ought to carry off. On the contrary, the waste, the poisonous waste of the different parts of the body through which the blood circulates, clings to the blue fluid, and coursing with it to other parts of the body, poisons them, and thus destroys health, pleasure, and life by its unwholesome potency.

Cycling is one of the best methods of oxydising the blood which modern science has discovered. To begin with, it carries men rapidly out of the city where oxygen is not, into the country where oxygen is: and in the next place it raises the rate of the breathing from fifteen or sixteen respirations in the minute to twenty-five or thirty a minute; and that in a natural and wholesome way. The lungs thus inhale at least three times the ordinary quantity of oxygen they would inhale in town; the waste products in the blood are thereby effectually destroyed, and are carted off by the pulmonary exhalations with the utmost rapidity and ease. The skin, moreover, which itself performs the functions of a huge lung in several important particulars, is roused to ten times its normal energy of action, and heaves out the waste products of the body, to use a familiar metaphor, by shiploads.

The conditions of health which have been thus cursorily described are well known to everybody. No one thinks of disputing them. All these conditions are fulfilled by intelligent cycling; and intelligent cycling is open to every man who has the use of his limbs, up to eighty years of age, and even longer. Cycling makes not only health, but happiness. It produces a happiness which one feels, and gains possession of at once, and retains. The cyclist, who has got his weeks of preliminary training over, never fails to feel exhilarated and bright within fifteen minutes of mounting his iron steed. The rapid movement, the warming blood, the expanding lungs, the rushing antagonism of the life-giving breezes, the sense of conscious power, all combine to make him feel how small have been the trivial cares of the day, and how glorious a thing it is to have a muscular and healthy body capable of delightful movement, and clear eyes and unclouded brain for the enjoyment of sky, and field, and tree, and river; and all the ten thousand active and restful forms of living and healthy nature. Undoubtedly, cycling is a discovery to the middle-aged man. It takes twenty years off his life at a stroke. It doubles his capacity for thought and work. The cycle has revolutionised the health of the youthful city man. It will do the same, and even more, for the man of middle age, if he will but try it and use it intelligently.

Cycling is one of the easiest arts in the world to learn. It is accompanied by practically no danger. Probably the man of forty-five will imagine that he would look very ridiculous if mounted on an iron steed, and that even the wife of his bosom might be inclined to laugh at him. But all such annoyances are obviated if a tricycle be used instead of a bicycle. Half an hour a day for a fortnight will convert a man of average intelligence into quite a skilled rider. Some people have ridden five or six miles without discomfort in their second or third trial. Whatever the timid or the worshippers of habit may say, it is quite certain that the tricycle or the bicycle for the middle-aged man of average bodily vigour is an unmixed good.

